# Precise Prediction of Calpain Cleavage Sites and Their Aberrance Caused by Mutations in Cancer

**DOI:** 10.3389/fgene.2019.00715

**Published:** 2019-08-08

**Authors:** Ze-Xian Liu, Kai Yu, Jingsi Dong, Linhong Zhao, Zekun Liu, Qingfeng Zhang, Shihua Li, Yimeng Du, Han Cheng

**Affiliations:** ^1^School of Life Sciences, Zhengzhou University, Zhengzhou, China; ^2^State Key Laboratory of Oncology in South China, Collaborative Innovation Center for Cancer Medicine, Sun Yat-sen University Cancer Center, Guangzhou, China; ^3^Lung Cancer Center, West China Hospital, Sichuan University, Chengdu, China; ^4^Institute of Life Sciences, Southeast University, Nanjing, China

**Keywords:** calpain, cleavage site, prediction, cancer mutation, deep learning

## Abstract

As a widespread post-translational modification of proteins, calpain-mediated cleavage regulates a broad range of cellular processes, including proliferation, differentiation, cytoskeletal reorganization, and apoptosis. The identification of proteins that undergo calpain cleavage in a site-specific manner is the necessary foundation for understanding the exact molecular mechanisms and regulatory roles of calpain-mediated cleavage. In contrast with time-consuming and labor-intensive experimental methods, computational approaches for detecting calpain cleavage sites have attracted wide attention due to their efficiency and convenience. In this study, we established a novel computational tool named DeepCalpain (http://deepcalpain.cancerbio.info/) for predicting the potential calpain cleavage sites by adopting deep neural network and the particle swarm optimization algorithm. Through critical evaluation and comparison, DeepCalpain exhibited superior performance against other existing tools. Meanwhile, we found that protein interactions could enrich the calpain-substrate regulatory relationship. Since calpain-mediated cleavage was critical for cancer development and progression, we comprehensively analyzed the calpain cleavage associated mutations across 11 cancers with the help of DeepCalpain, which demonstrated that the calpain-mediated cleavage events were affected by mutations and heavily implicated in the regulation of cancer cells. These prediction and analysis results might provide helpful information to reveal the regulatory mechanism of calpain cleavage in biological pathways and different cancer types, which might open new avenues for the diagnosis and treatment of cancers.

## Introduction

With a nucleophilic cysteine at the catalytically active site, calpains (calcium-activated non-lysosomal proteases) are an important evolutionarily well-conserved family of Ca^2+^-dependent cysteine proteases (Croall and Ersfeld, 2007; Ono and Sorimachi, 2012). In mammalians, calpains are diffusely expressed, and there are approximately 16 known genes of the calpain superfamily at present, among which the calpain 1 (μ-calpain) and calpain 2 (m-calpain) are the most well-studied isoforms called the “conventional” calpains (Goll et al., 2003; Franco and Huttenlocher, 2005; Croall and Ersfeld, 2007). Through cleaving various substrates, calpains play a pivotal role in a wide range of cellular and physiological processes, such as the regulation of embryogenesis, differentiation, signal transduction, apoptosis, and necrosis as well as remodeling of cytoskeletal attachments in the process of cell migration and cell cycle progression (Schoenwaelder et al., 1997; Squier et al., 1999; Glading et al., 2002; Franco and Huttenlocher, 2005; Tan et al., 2006; Croall and Ersfeld, 2007). Moreover, there are numerous studies have indicated that aberrant activities of calpains are closely related to a variety of diseases and cancers, including neurodegeneration, limb girdle muscular dystrophies, type II diabetes, and tumorigenesis (Branca, 2004; Tompa et al., 2004; Bertipaglia and Carafoli, 2007; Storr et al., 2011). Therefore, the identification of precise cleavage sites in calpain substrates is fundamental for dissecting the exact molecular mechanisms and calpain function.

Current experimental approaches for the identification of calpain cleavage sites mainly include Edman N-terminal sequencing, mass spectrometry, and a peptide library approach; thus, a large number of calpain cleavage proteins and sites have been experimentally verified. The database CaMPDB (Duverle et al., 2010), which contains calpains, substrates, and cleavage sites as well as upstream inhibitors, has been constructed based on information extracted from the literature. Although the application of current experimental techniques has increased the number of experimentally identified calpain substrates with cleavage sites, there are also numerous substrates and cleavage sites that remain to be discovered. Moreover, the identification and characterization of calpain substrates and cleavage sites by experiments are usually expensive, time-consuming, and laborious. Therefore, the computational approaches developed to accurately predict calpain substrates and cleavage sites may complement and guide the experimental studies to promote the discovery of putative cleavage sites.

In 2004, Tompa et al. (2004) collected 49 calpain substrates with 106 cleavage sites from the literature and studied the preferences of amino acid residues around cleavage sites. Then, a position-specific scoring matrix (PSSM) was generated to predict potential cleavage sites, while the preferred amino acids for μ-calpain and m-calpain recognition were identified as Leu, Thr, and Val residues in the P_2_ position and Lys, Tyr, and Arg residues in the P_1_ position. Using this information, an online tool called PoPS (Boyd et al., 2005), which allows the users to build their computational models of protease specificity based on their own training data, was developed. Through the scoring methods of frequency and substitution matrix, Verspurten et al. (2009) developed SitePrediction to predict the potential cleavage sites of proteinase substrates. In 2011, DuVerle et al. (2010, 2011) built an online resource CaMPDB, which also provided a cleavage site prediction tool for calpains, and then, the calpain cleavage prediction was further updated by adopting the approach of multiple kernel learning. Based on the GPS (group-based prediction system) algorithm (Xue et al., 2008), the *in silico* prediction tool GPS-CCD (Liu et al., 2011) was developed to predict potential cleavage sites for calpain, which provided both of the online service and local software packages. Later, Fan et al. (2013) constructed the LabCaS program for the prediction of the calpain-specific cleavage sites based on conditional random fields algorithm. These computational approaches can be roughly classified into two categories: 1) methods based on sequence alignment, including PoPS (Boyd et al., 2005), SitePrediction (Verspurten et al., 2009), and GPS-CCD (Liu et al., 2011); and 2) methods based on machine learning, including CaMPDB (Duverle et al., 2010; Duverle et al., 2011) and LabCaS (Fan et al., 2013). Generally speaking, sequence alignment-based tools use the amino acid substitution matrix to calculate the similarity score between two sequences, whereas the approaches based on machine learning extract the features from sequence by feature engineering and then select a machine-learning algorithm to build the model. To date, although a number of predictors with good prediction performance have been developed, the main limitations of these methods are that sequence alignment-based approaches rely on the amino acid substitution matrix to achieve the best result and the machine learning-based approaches depend heavily on data preprocessing and feature selection.

To provide a promising and credible solution, deep learning method was applied in our work (Lecun et al., 2015; Li et al., 2019). As a branch of machine learning, deep learning has overcome some key issues and can learn complex features through a combination of simpler features extracted from sequence. To date, deep learning techniques have been adopted in a number of bioinformatics studies, such as biological sequence analysis. For example, DeepBind (Alipanahi et al., 2015) was developed to predict the sequence specificities of RNA- and DNA-binding proteins based on convolutional neural network (CNN). By combining CNN with a two-dimensional attention mechanism, MusiteDeep (Wang et al., 2017) was constructed for the general and kinase-specific phosphorylation sites prediction. Recently, Xie et al. (2018) established a computational tool called DeepNitro by combining primary sequence features and deep neural network (DNN) for predicting protein nitration and nitrosylation sites. Compared with the traditional machine learning-based methods, these approaches have reached a better prediction performance with the same features. However, to date, an available deep-learning framework for calpain cleavage site prediction is still lacking.

Inspired by the research of Li et al. (2018) and Zou et al. (2018), here, we present a novel deep-learning framework named DeepCalpain for predicting calpain cleavage sites. First, 442 experimentally identified cleavage sites in 169 proteins with 176 cleavage sites for μ-calpain and 256 cleavage sites for m-calpain were collected. Then, we extracted four effective features from the query sequences, including amino acid composition (AAC), binary encoding profiles (BE), PSSM, and composition of k-spaced amino acid pairs (CKSAAP). These abstracted sequence features of calpain cleavage sites were then integrated with DNN to construct the predictor, while the particle swarm optimization (PSO) algorithm was adopted to optimize the hyperparameters of the model. Moreover, 4-, 6-, 8-, and 10-fold cross-validations of the training data demonstrated acceptable performance and robustness of the prediction system. By comparison, DeepCalpain outperformed other existing tools. In addition, we comprehensively analyzed calpain cleavage associated mutations across 11 cancers with the help of DeepCalpain to reveal the regulatory roles of calpain-mediated cleavage in biological pathways and cancer development and progression, which will provide certain help for the diagnosis and treatment of cancer.

## Methods

### Data Collection and Preparation

We searched the literatures from PubMed (http://www.ncbi.nlm.nih.gov/pubmed) to retrieve the published experimentally identified substrates with cleavage sites for calpain using the keyword “calpain.” The sequence of each protein was retrieved from the UniProt database (UniProt Consortium, 2015), and the exact cleavage positions were noted. After removing redundant data, 442 unique cleavage sites in 169 proteins were finally obtained, including 176 cleavage sites for μ-calpain and 256 cleavage sites for m-calpain (**Table S1**).

With the calpain cleavage sites in the center and surrounded by 15 residues of upstream and downstream, we generated the calpain cleavage peptides for feature extraction. All experimentally identified cleavage sites were regarded as the positive dataset, whereas all sites that were not cleavable in the same proteins were taken as the negative dataset. In total, we obtained 442 positive sites and 160,698 negative sites in 169 proteins for training.

### Feature Extraction

#### Amino Acid Composition

AAC (Radivojac et al., 2010; Lee et al., 2011) is an elementary feature and describes the occurrence frequency of the 20 native amino acids (ACDEFGHIKLMNPQRSTVWY) in a protein sequence. To ensure the calpain cleavage peptides with the same length (30 amino acids), we added one or more “-” characters to them, so the AAC dimension is 21 in our work.

#### Binary Encoding Profiles

Binary encoding (Song et al., 2010) is an encoding scheme that was developed from the binary language of computer. We transformed the substrate sequences into *n*-dimensional vectors. As mentioned above, the “-” character was added to represent the pseudo amino acid at the N- or C-terminus of proteins. Therefore, 21 types of amino acids are composed of ACDEFGHIKLMNPQRSTVWY-. Then, each amino acid is represented by a 21-dimensional binary vector, such as the amino acid A corresponding to (100000000000000000000), the amino acid C corresponding to (010000000000000000000), and the ”–” character corresponding to (000000000000000000001). In this regards, the dimension of BE coding for each calpain cleavage peptide is 630.

#### Position-Specific Scoring Matrix

In biological analysis, evolutionary conservation as an essential factor should be considered. Through adopting all training data as the background, we obtained the occurrence frequency of the amino acid at each position to generate a calpain cleavage-specific matrix rather than employing the scoring matrix of blosum62 or others. For a new peptide, we use the formula below:

$$\{\begin{array}{c} {\text{f}}_{1}=P\left({\mathrm{AA}}_{1}\right)\\ {\text{f}}_{2}=P\left({\mathrm{AA}}_{2}\right)\\ \vdots \;\vdots \\ {\text{f}}_{31}=P\left({\mathrm{AA}}_{31}\right)\\ {\text{f}}_{32}=N\left({\mathrm{AA}}_{1}\right)\\ {\text{f}}_{33}=N\left({\mathrm{AA}}_{2}\right)\\ \vdots \;\vdots \\ {\text{f}}_{62}=N\left({\mathrm{AA}}_{31}\right) \end{array}$$

In this formula, the calpain cleavage peptides extracted from their protein sequences are composed of 31 amino acids, while the P(R_1_) element represents the occurrence frequency of amino acid residue AA_1_ in position 1 of the positive group, the P(R_2_) element represents the occurrence frequency of AA_2_ in position 2 of the positive group, the N(R_1_) element indicates the occurrence frequency of AA_1_ in position 1 of the negative group, the N(R_2_) element denotes the occurrence frequency of AA_2_ in position 2 of the negative group, and so on. The dimension of this PSSM profile is 60.

#### Composition of K-Spaced Amino Acid Pairs

Since the relative position of the amino acids in a protein may affect the function of this protein, the features of correlation and dependence for amino acids surrounding calpain cleavage sites are helpful for the prediction. In this work, the CKSAAP (Zhao et al., 2012) was employed to extract the amino acids order information of protein sequences. The detailed processes of CKSAAP are described as follows. Given that the extracted peptides from protein sequences are composed of 21 types of amino acids in this work, there are 441 possible types (*AA, AC, AD*, …, –) of amino acid pairs with 0-, 1-, 2-… or k-space (i.e., the pairs are separated by *0-, 1-, 2-*,…, or *k* amino acids) for a peptide including 2n+1 amino acids. Then, a feature vector is adopted to describe the composition of these amino acid pairs, which is generated by CKSAAP defined as (*N_AA_*, *N_AC_*, *N_AD_*, *…, N*_−−_)_441_. The value of each feature in this vector indicates the occurrence frequency of a corresponding amino acid pair in the extracted peptide. For example, if the amino acid pair of AC occurs *m* times in a peptide, the value of *N* *_AC_* in the vector is equal to *m* and so forth. As the *k* value increased, although there would be a growing tendency of the accuracy and sensitivity for the prediction model, the computational time and cost of the DNN model training would also drastically increase. In this regard, the CKSAAP encoding with the *k* value equal to 0, 1, 2, and 3 was merely considered in this work; so, the total dimension of the feature vector with 3-space is 441 × (3 + 1) = 1764.

#### Deep Neural Network Models for Prediction

**Figure 1:**
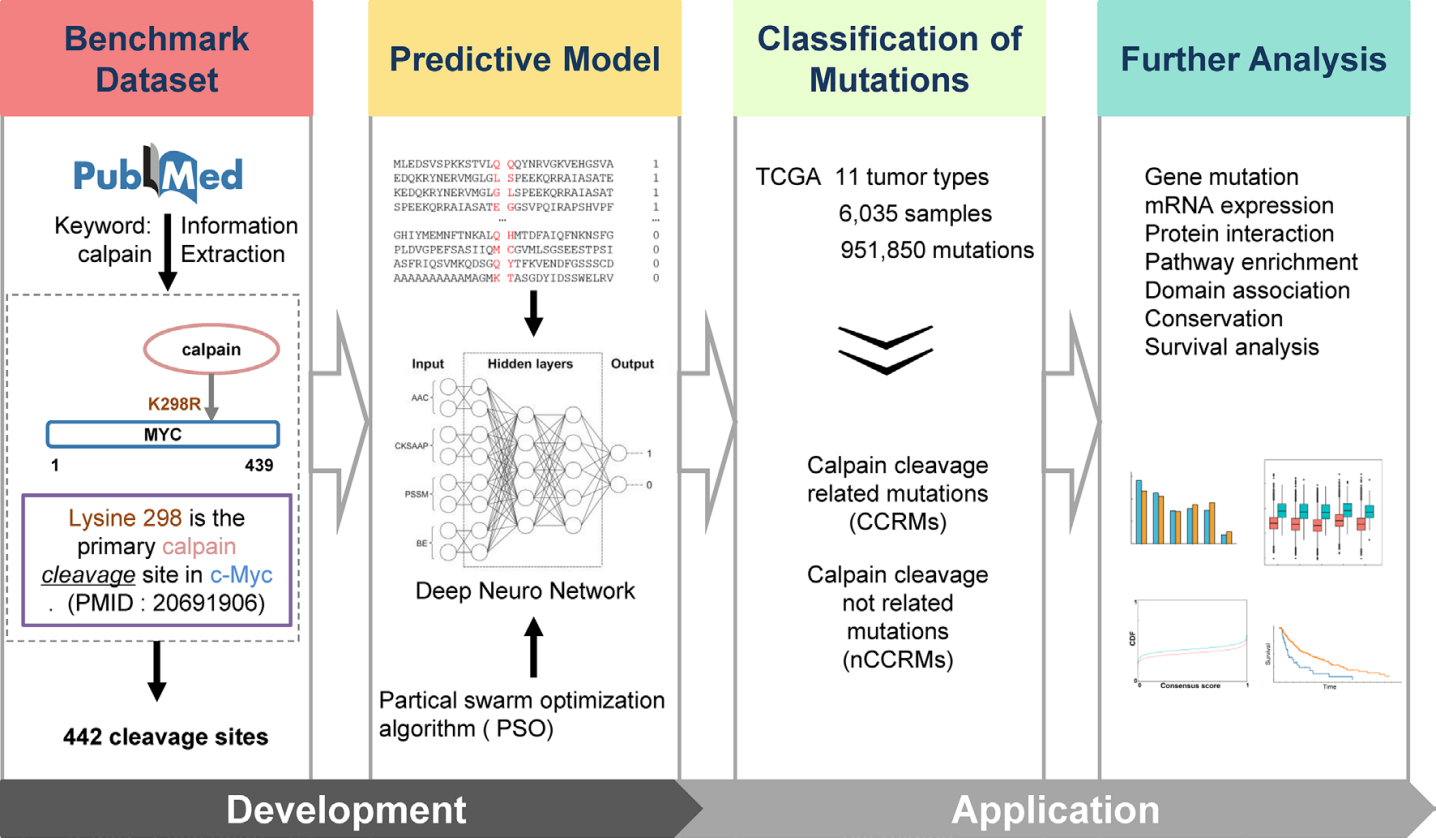
Overall methodology. Highlighted are experimentally identified calpain cleavage sites extracted from PubMed by text mining; multi-network deep-learning and PSO algorithm; and the integrative analysis of the connections between calpain-mediated cleavage and cancers.

For the detection of potential calpain substrates and cleavage sites, a deep neural network model was introduced into our prediction algorithm. The model architecture was presented in **Figure 1**. For a given protein sequence, the cleavage sites are extracted with a peptide length of 30 comprising a central calpain cleavage amino acid pair and 15-residue flanking at each side. Based on the sequences, the protein fragments are coded through four feature extraction methods. The sequences are transferred into the input format of this model; then, the DeepCalpain software predicts whether the residue can be cleaved by calpain. The DeepCalpain model consists of three main components, including the input layer, the hidden layers, and the output layer. The input layer contains four submodules to store the features extracted through the methods of AAC, BE, PSSM, and CKSAAP. In each submodule, the input data are trained in the hidden layer. Then, after sufficiently learning the features, the four submodules are merged and flattened into a fully connected layer. In the end, it can be simply formulated as a binary classification problem for the prediction in the output layer, while the two-dimensional result represents the probability of calpain cleavage. The sum of the two probabilities equal one, so only the probability of calpain cleavage is considered as the score for the input peptide. Moreover, to generate the optimal performance, the PSO algorithm was integrated and the python package pyswarm (https://github.com/tisimst/pyswarm) was adopted to optimize the hyperparameters. To avoid overfitting, we applied dropout to make sure the positive data not be over-represented as previous study described (Xia et al., 2018; Umarov et al., 2019), the dropout rate is determined by PSO. The detailed parameters of the model adjusted by PSO were displayed in **Table S3**.

#### Performance Evaluation

As previously described (Liu et al., 2012), three measurements, including sensitivity (*Sn*), specificity (*Sp*), and accuracy (*Ac*), were adopted to estimate the prediction performance of DeepCalpain. The detailed description of these three measurements was defined as below:

$$\mathrm{Sn}=\frac{\mathrm{TP}}{\mathrm{TP}+\mathrm{FN}}$$

$$\mathrm{Sp}=\frac{\mathrm{TN}}{\mathrm{TN}+\mathrm{FP}}$$

$$\mathrm{Pr}=\frac{\mathrm{TP}}{\mathrm{TP}+\mathrm{FP}}$$

We performed 4-, 6-, 8-, and 10-fold cross-validations to evaluate the robustness of this prediction system, while the receiver operating characteristic (ROC) curves and AUCs (area under ROCs) were drawn and calculated. In addition, 5-fold cross-validation was also performed to demonstrate the superiority of DeepCalpain in comparison with several other existing tools.

#### Calpain Cleavage–Related Mutation Analysis

TCGA somatic mutation data of 11 cancer types (BLCA, BRCA, CESC, COAD, HNSC, LIHC, LUAD, LUSC, SKCM, STAD, and UCEC) were downloaded from Xena Browser (https://xenabrowser.net/datapages/). The redundant mutations in the same patients were removed, and only missense variants were retained for further analysis. Based on the prediction results of DeepCalpain, we further classified the mutations into two types based on whether calpain-mediated cleavage was affected.

#### Differentially Analysis of Calpain Expression

Pan-cancer mRNA expression level 3 normalized data were downloaded from Firehose (http://gdac.broadinstitute.org). After calculating the fold change and adjusted p-value by the R package DESeq2, we defined the genes with an adjusted *p*-value less than 0.05 as differentially expressed genes.

#### KEGG Enrichment Analysis

To better understand the potential function of calpain cleavage–related mutation sites and proteins, the enrichment analysis of these proteins in KEGG pathways was performed using KOBAS (Xie et al., 2011). The visible network was constructed using Cytoscape (Ono et al., 2014).

#### Evolutionary Conservation Analysis

The sequence evolutionary conservation score of each missense mutation stored in the phastCons score profile was calculated in ANNOVAR (Siepel et al., 2005). It adopts phylogenetic hidden Markov model (phylo-HMM) to quantitatively measure the nucleotide substitution probability for each site in the genome, and we could extract the evolutionary conservation level for each mutation site with the phastCons score profile. In this study, we used the phastCons score to represent the evolutionary conservation scores of all mutations, and then plotted the cumulative distribution fraction (CDF) curve to evaluate the difference between calpain cleavage–related mutations and other mutations.

#### Survival Analysis

The clinical data of 11 cancer types were downloaded from Xena Browser (https://xenabrowser.net/datapages/) for further analysis, while the R package “survival” (https://cran.r-project.org/web/packages/survival/) was adopted to acquire the overall survival through Kaplan-Meier estimation. To clarify the relations between calpain cleavage–related mutations (CCRM) and survival, we classified the patients into two groups, including patients with less than six CCRMs and patients with six or more CCRMs. Then, we employed the log-rank test to compare the survival of the two patient groups. The number 6 was determined to balance the patient size of the two groups and choose as few CCRMs as possible.

#### Implementation of the Webserver

The online service of DeepCalpain was implemented in Python + PHP and is freely available at http://deepcalpain.cancerbio.info. Moreover, IUPred (Dosztanyi et al., 2005) and NetSurfP (Petersen et al., 2009) softwares were also integrated to predict the protein structural features, including disorder regions, surface accessibilities, and secondary structures. The experimentally identified protein-protein interaction (PPI) dataset were downloaded and integrated from BioGRID (Chatr-Aryamontri et al., 2015), BioPlex (Huttlin et al., 2015), IID (Kotlyar et al., 2016), I2D (Brown and Jurisica, 2007), and IntAct (Orchard et al., 2014). In total, there were 309,321 PPIs in 20,379 proteins that were obtained, and the visible network was constructed by Cytoscape (Ono et al., 2014). To provide a robust service, we tested the website of DeepCalpain on a variety of web browsers, such as Internet Explorer, Google Chrome, and Mozilla Firefox. It will take 40 s for the default protein in average. When user submits more than one protein, DeepCalpain will predict and show the first protein as default. Users can select which protein to display in the result page, and this will take 20 s in average.

## Results

### Development of DeepCalpain for the Prediction of Calpain Cleavage Sites

The experimentally confirmed calpain cleavage sites were retrieved through keywords “calpain” from the scientific literature (**Figure 1**). After redundancy removal, we finally obtained 442 experimentally identified calpain cleavage sites in 169 proteins, which contained 176 μ-calpain cleavage sites and 256 m-calpain cleavage sites (**Table S1**). For the preparation of training dataset, the known calpain cleavage sites were taken as the positive dataset, while all other non-cleavable sites in the same proteins were regarded as the negative dataset. In total, the non-redundant training dataset of calpain cleavage contained 442 positive sites and 160,698 negative sites in 169 proteins. Then, we developed DeepCalpain software for the prediction of calpain cleavage sites based on multi-network deep learning and PSO algorithm. Four protein sequence features, including AAC, PSSM, BE, and CKSAAP, were used to extract the sequence features (**Figure 1**). The online service of DeepCalpain was implemented in Python and PHP, while two *in silico* tools IUPred (Dosztanyi et al., 2005) and NetSurfP (Petersen et al., 2009) were also integrated to predict the structural features of proteins, including disorder regions, surface accessibilities, and secondary structures. Furthermore, the mutations downloaded from TCGA database were analyzed by DeepCalpain and then classified into two types, including CCRMs and calpain cleavage non-related mutations (nCCRMs). A series of analyses were further performed, including gene mutation, mRNA expression, protein interaction, pathway enrichment, domain association, conservation, and survival analyses (**Figure 1**).

### The Sequence and Structure Preferences of Calpain Cleavage Sites

**Figure 2:**
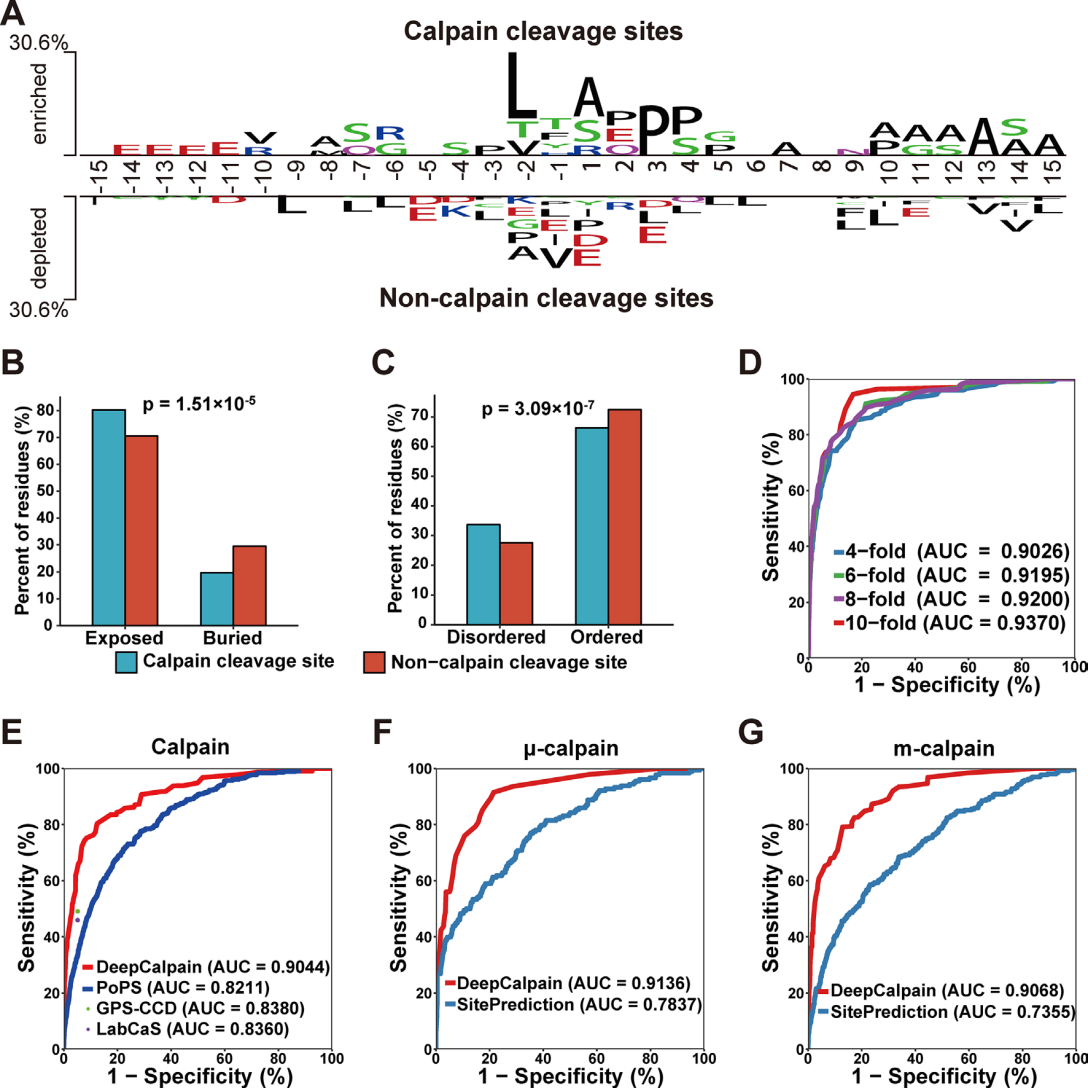
**(A)** The preferences for the amino acids around the calpain cleavage sites and non-calpain cleavage sites. **(B–C)** Comparison of the surface accessibility **(B)** and disorder information between calpain cleavage sites and non-calpain cleavage sites. **(D)** The 4-, 6-, 8-, and 10-fold cross-validations results for calpain. **(E–G)** Comparison of the models of calpain **(E)**, µ-calpain **(F)**, and m-calpain **(G)** with the existing tools, and the dots were the *Sn* and *Sp* values adopted from the related literatures.

Using the collected calpain cleavage sites, the sequence features were analyzed through the software Two Sample Logo (Vacic et al., 2006). The difference between calpain cleavage sites and non-calpain cleavage sites were shown in **Figure 2A**. Leucine was enriched at the -2 position in calpain cleavage sites and enriched at the -9, -7, -6, -3, +3, +4, +5, + 6, + 9, +10, and +15 positions in non-calpain cleavage sites. Another hydrophobic amino acid alanine was enriched at the -8, +1, +7, +10, +11, +12, +13, +14, and +15 positions in calpain cleavage sites and enriched at -2 position in non-calpain cleavage sites, while proline was also enriched at the -3, +3, and +4 positions in calpain cleavage sites and enriched at -2 and +1 positions in non-calpain cleavage sites (**Figure 2A**). Collectively, it was suggested that the hydrophobic residues around the cleavage site were preferred by calpains. To further explore the recognition preferences of calpain cleavage, comparative analysis on structural features of the calpain cleavage sites and the non-calpain cleavage sites were performed. The calpain cleavage sites were enriched in surface exposed residues (*p*-value = 1.51*10^-5^, two proportions z-test) (**Figure 2B**) and dramatically occurred in disordered regions (*p*-value = 3.09*10^-7^, two proportions z-test) (**Figure 2C**).

### Performance Evaluation and Comparison

Based on the features extracted by AAC, BE, PSSM, and CKSAAP, we constructed DeepCalpain for calpain cleavage sites prediction. To evaluate the prediction performance and robustness of DeepCalpain, the 4-, 6-, 8-, and 10-fold cross-validations of the training dataset were performed. For each validation, the measurements of *Sn*, *Sp*, and *Ac* were calculated. The ROC curves were drawn, while the values of the AUC were 0.9026 (4-fold), 0.9195 (6-fold), 0.9200 (8-fold), and 0.9370 (10-fold), respectively (**Figure 2D**). Due to the results of different validations were very similar with each other, DeepCalpain is a stable and robust predictor.

To demonstrate the superiority of DeepCalpain, we compared its prediction performance with several existing tools, including GPS-CCD (Liu et al., 2011), LabCaS (Fan et al., 2013), and PoPS (Boyd et al., 2005) (**Figure 2E**). We evaluated the prediction performance using 5-fold cross-validations, and the dots outside of the lines were adopted from previous publications of GPS-CCD and LabCaS to perform comparison. The AUC values for DeepCalpain and PoPS were 0.9044 and 0.8211, respectively. In addition, the AUC values were 0.8380 for GPS-CCD and 0.8360 for LabCaS, which indicated that DeepCalpain significantly outperformed the existing tools. And besides, we exhaustively tested the flanking residues around the cleavage sites and found that the AUC did not change significantly when the cleavage sites were surrounded by more than 10 residues upstream and downstream (**Figure S1**, **Table S2**), which suggested 15 amino acids flanking size is enough for DeepCalpain to gain an excellent performance.

The concept of transfer learning was adopted to build the calpain-specific models from the pretrained calpain cleavage model to solve the small-sample problem in calpain-specific cleavage site prediction, and the submodels of μ-calpain and m-calpain were constructed, respectively. The parameters of each model were displayed in **Table S3**. To further illustrate the performance superiority of DeepCalpain, the submodels of μ-calpain and m-calpain were compared with SitePrediction (Verspurten et al., 2009). For the μ-calpain, the 5-fold cross-validation AUC value of DeepCalpain was 0.9136, revealing a better performance than SitePrediction (AUC = 0.7837) (**Figure 2F**). Using a similar situation, the AUC value of DeepCalpain for m-calpain (0.9068) was significantly higher than that of SitePrediction (0.7355) (**Figure 2G**). Taken together, these results demonstrated that the performance of DeepCalpain was superior to the previously reported predictors.

### Connections Between Cancer Mutations and Calpain Cleavage Sites

**Figure 3:**
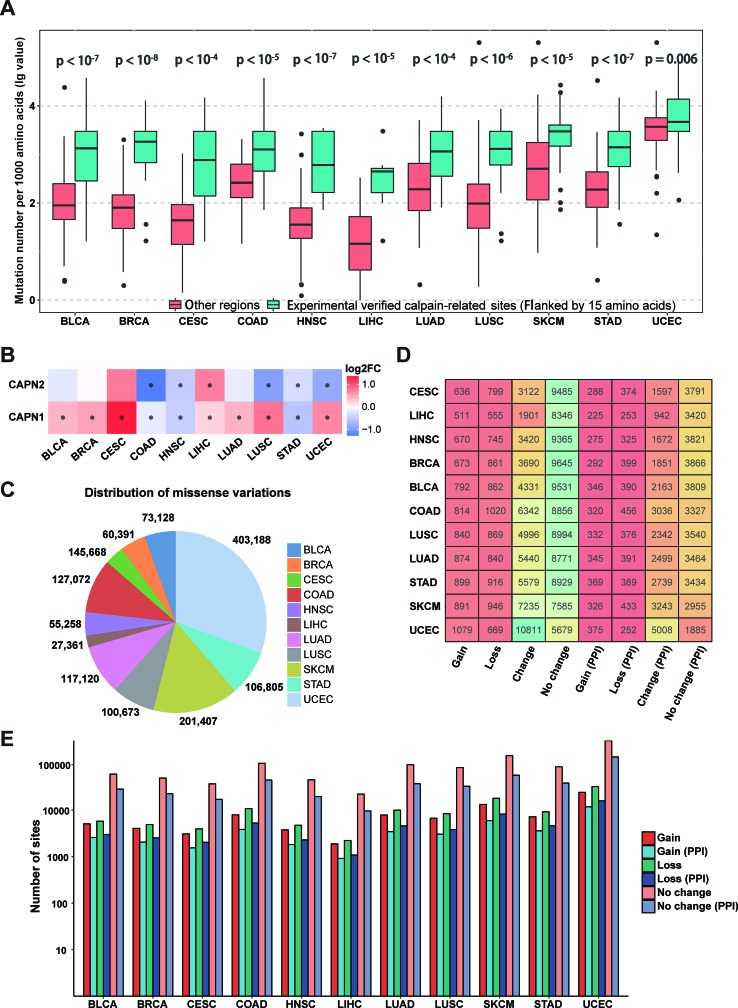
Connections between calpain cleavage sites and genetic variants. **(A)** Mutations preferentially occur at the regions around calpain cleavage sites. **(B)** Calpains were differential expressed across cancers. **(C)** Summary of the distribution of missense variations across cancers. **(D)** Summary of proteins cleavage aberrant triggered by mutations. **(E)** Summary of mutation site types across cancers.

Since calpain-mediated cleavage is significantly associated with a variety of pathological phenomena from neurodegeneration to cancers (Branca, 2004; Bertipaglia and Carafoli, 2007; Storr et al., 2011), the somatic mutations in different cancer types might alter calpain-mediated regulatory signaling pathways. To investigate specific associations between calpain cleavage sites and cancer mutations, the 951,850 somatic mutations from 6,035 samples across 11 cancer types (BLCA, BRCA, CESC, COAD, HNSC, LIHC, LUAD, LUSC, SKCM, STAD, and UCEC) were collected from TCGA and mapped to the experimentally verified calpain-related proteins. Then, the functional impact of somatic mutations on calpain cleavage sites was researched through the bootstrap test (Chen et al., 2018). Obviously, the somatic mutations more preferably occurred at the experimentally verified calpain cleavage sites (flanked by 15 amino acids) than other regions among the 11 cancer types (**Figure 3A**). The mRNA expression data indicated that μ-calpain and m-calpain were differentially expressed across cancers, while the majority was upregulated (**Figure 3B**). This phenomenon is consistent with previous studies, which reported that increased μ-calpain and m-calpain expression were observed in numerous cancers, such as schwannomas, meningiomas, colorectal adenocarcinomas, and breast cancer (Kimura et al., 1998; Lakshmikuttyamma et al., 2004; Storr et al., 2011; Storr et al., 2012). Generally, the above analysis suggested that calpain-mediated cleavage was highly associated with cancers, and the genetic variation in cancers might alter the calpain-mediated regulatory network.

Previous research indicated the substrates of calpain cleavage were enriched in the proteins that interact with calpains (Duverle et al., 2010). We integrated several protein interaction databases and found that not only direct interactions (Ratio = 22.22, *p*-value = 1.26*10^-18^) but also sharing interacting proteins (ratio = 2.15, *p*-value = 3.16*10^-16^) could significantly enrich calpain-mediated cleavage regulatory relationships. Thus, we presented the protein interaction network for calpains and the substrates in the predictor to provide helpful information.

To further characterize the relationships between calpain-mediated cleavage and cancer mutations, we analyzed the aberrant calpain-mediated cleavage affected by missense variations across 11 cancer types. Among all selected cancers, UCEC has the largest number of missense variations (403,188), whereas LIHC carries the fewest number of missense variations (27,361) (**Figure 3C**). Furthermore, we generated peptide windows composed of 30 amino acids with a cleavage site in the middle flanked with 15 amino acids upstream and downstream. Each site in a protein would generate two peptides: one was extracted from the origin sequence, and the other was from the new sequence after mutation. Finally, we predicted the cleavage probability for these sequence windows before and after mutation based on DeepCalpain. All missense variations were classified into four types by comparing the change of calpain cleavage status for the original and mutated proteins. i) Gain indicates that a missense variation event creates one or multiple calpain cleavage sites; ii) loss indicates that the presence of a missense variation disrupts all calpain cleavage sites; iii) change indicates that a missense variation changes the position of calpain cleavage site; and iv) no change indicates that the missense variation event has no effect on the status of the calpain cleavage. Then, the distribution of proteins across cancer types influenced by these four types of missense variations were counted and shown in **Figure 3D**. From the results, it is evident that the positions of calpain cleavage sites in a large number of proteins were changed, and greater than 43.9% of the cleaved substrates were influenced on average. Additionally, the numbers of proteins influenced by each type of missense variations varied remarkably among the 11 cancers, especially in UCEC, which amounted to 10,811 proteins with altered calpain cleavage sites (**Figure 3D**). Since the PPI information could enrich calpain-mediated cleavage regulatory network, we filtered the potential substrates with PPI information. The numbers of target proteins affected by each type of missense variation in each selected cancer type were reduced to approximately one-third, whereas the proportions were generally similar (**Figure 3D**). Moreover, the numbers of calpain cleavage sites influenced by these types of missense variations across 11 cancers with or without PPI information were also tallied (**Figure 3E**). Taken together, the above results suggested that abnormal modification of calpain cleavage is heavily implicated in the regulation of cancer cells.

### Functional Analysis of Calpain Cleavage–Related Mutations

**Figure 4:**
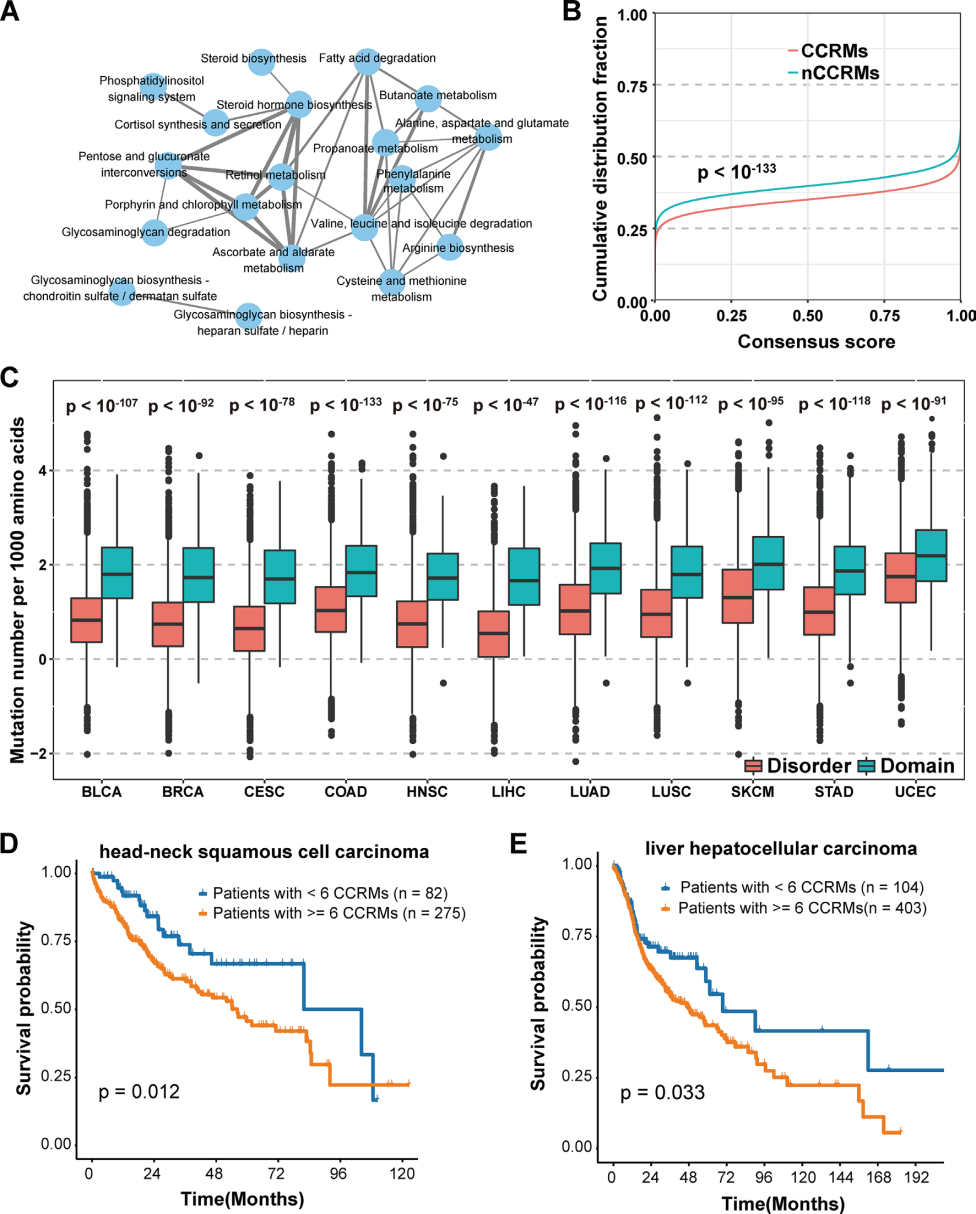
Systematic analysis of the impact of calpain cleavage–related mutation sites and proteins. **(A)** CCRM proteins were significantly enriched in cancer-related pathways. **(B)** CCRM sites were more conserved than other mutations. **(C)** CCRM sites showed a preference to be enriched in known functional domain regions. **(D–E)** Patients with at least six CCRM sites had significantly worse clinical prognostic in HNSC **(D)** and LIHC **(E)**.

We further classified the mutations into two categories based on whether calpain-mediated cleavage was affected by the mutation. Mutations lead to gain or loss of calpain cleavage were classified into calpain cleavage–related mutation (CCRM) category, while the others were in the nCCRM category. And the proteins with CCRM sites were regarded as CCRM proteins. To better understand the potential function of CCRM sites and proteins, enrichment analysis of these proteins in KEGG pathways was performed using KOBAS (Xie et al., 2011). Compared with all mutated proteins, the CCRM proteins were significantly enriched in several pathways related to metabolism, degradation, and biosynthesis, such as butanoate metabolism, glycosaminoglycan degradation, and steroid hormone biosynthesis (**Figure 4A**). According to the previously published reports, these enriched pathways were involved in cancer cell proliferation and apoptosis (Afratis et al., 2012; Donohoe et al., 2012; Mostaghel, 2013), suggesting our identified CCRM proteins play critical roles during tumorigenesis.

In addition to KEGG pathway analysis, the evolutionary conservation of CCRM sites was also performed. Through the 100-way scheme of phastCons score calculated in ANNOVAR (Siepel et al., 2005), we extracted the conservation score for each mutation, including both calpain cleavage–related mutations and the other missense variations. Compared with nCCRMs, CCRMs were more conserved (*P* value < 10^-133^, Kolmogorov-Smirnov test) (**Figure 4B**), indicating that these mutations may be driven by stronger positive selection during cancer progression. To gain further insights into the impacts of CCRM on protein functions, the bootstrap test was performed and it was found that CCRMs preferentially located at the functional domains than disordered regions among the 11 cancer types, which suggested that these mutations may affect protein functions in different types of cancer (**Figure 4C**). And besides, we found that the C > T mutation pattern was enriched in CCRMs (**Figure S2A**). Furthemore, it was observed that CCRMs had higher VAF values than nCCRMs (**Figure S2B**), which meant that these mutations might be more functional in cancer. Taking the above analyses into consideration, we concluded that calpain cleavage–related mutations may under stronger positive selection during tumorigenesis and may play important roles in regulating cancer-related hallmarks and pathways.

Moreover, the clinical application of calpain-mediated cleavage regulatory relationship was largely unknown. To explore the clinical significance of CCRM sites, we classified the patients into two groups based on the presence of at least six CCRMs across 11 cancers (to balance the number of patients in both groups and select as few CCRMs as possible, the number of CCRMs was chosen to be six). Among the 11 cancer types, the patients with greater than or equal to six CCRMs had significantly poorer overall survival in head-neck squamous cell carcinoma (HNSC) (**Figure 4D**) and liver hepatocellular carcinoma (LIHC) (**Figure 4E**). The significant relationships between CCRMs and clinical prognosis found in these tested cancer types demonstrated that calpain-mediated cleavage crucially impacts the survival of patients with cancers.

## Discussion

As a widespread post-translational modification of proteins, calpain-mediated cleavage regulates a broad range of cellular events, such as proliferation, differentiation, cytoskeletal reorganization, and apoptosis (Schoenwaelder et al., 1997; Squier et al., 1999; Glading et al., 2002; Franco and Huttenlocher, 2005; Tan et al., 2006; Croall and Ersfeld, 2007). The identification of new substrates that undergo calpain cleavage in a site-specific manner is the necessary foundation for understanding the exact molecular mechanisms and regulatory roles of calpain-mediated cleavage. At present, although many studies have experimentally identified numerous calpain substrates with cleavage sites, there are also large-scale substrates and cleavage sites that have still not been detected. In contrast to time-consuming and labor-intensive experimental methods, the computational approaches for detecting calpain cleavage sites have attracted wide attention due to their efficiency and convenience. To the best of our knowledge, there are multiple tools that have been developed to predict the calpain cleavage sites, such as PoPS (Boyd et al., 2005), SitePrediction (Verspurten et al., 2009), GPS-CCD (Liu et al., 2011), and LabCaS (Fan et al., 2013). Nevertheless, many problems remained in these algorithms. Recently, the application of deep learning in machine learning algorithm has appeared as an important topic. In this work, a novel predictor DeepCalpain based on deep neural network in combination with PSO algorithm was presented. The 4-, 6-, 8-, and 10-fold cross-validations of the training dataset demonstrated that DeepCalpain is a stable and robust predictor system. Compared with other existing tools, DeepCalpain exhibited superior performance for the prediction of calpain cleavage sites. In addition, based on the transfer learning method, the submodels for the prediction of μ-calpain and m-calpain specific cleavage sites were also constructed, which outperformed existing approaches. Generally speaking, the deep learning-based predicting tool DeepCalpain is a useful program for detecting potential calpain cleavage sites, and the computational predictions followed by experimental validations would provide important hints for the further understanding of calpain-mediated cleavage mechanisms.

Previous studies have shown that calpain-mediated cleavage is highly correlated with cancer development and progression. For example, calpain-mediated cleavage of β-Catenin is important in prostate and mammary tumor cells (Rios-Doria et al., 2004), while calpain-mediated Myc cleavage promotes the survival of cancer cells (Conacci-Sorrell et al., 2014). To understand the regulatory mechanism of calpain-mediated cleavage in different cancer types at the systemic level, a series of analysis were performed in this study. By statistical analysis, we found the somatic mutations were significantly enriched in the regions around the calpain cleavage sites compared with other regions, while the μ-calpain and m-calpain mRNA expression levels were differentially expressed across 11 cancer types, which was consistent with previous analyses (Kimura et al., 1998; Lakshmikuttyamma et al., 2004; Storr et al., 2011; Storr et al., 2012). Moreover, to explore the potential functions of CCRM sites and proteins in different cancer types, we used the newly developed tool DeepCalpain to predict the cleavage potential for all variations and then classified them into four types, including Gain, Loss, Change, and Not changed according to their impact on calpain cleavage. From the results, we found that approximately 43.9% proteins undergoing calpain cleavage were influenced by missense variations on average across all selected cancers, which demonstrated that the abnormal modification of calpain cleavage plays an essential role in the development and progression of cancer cells. In addition, the KEGG enrichment analysis of CCRM proteins indicated that the pathways related to the processes of metabolism, degradation, and biosynthesis exhibited statistically significant enrichment in CCRM proteins. In addition, analyses of CCRMs demonstrated that CCRM sites were more conserved and had higher variant allele fraction (VAF) values than nCCRMs and significantly enriched in the domain regions, suggesting a potential positive selection and important function during cancer progression. Finally, we also observed that the CCRM sites were highly associated with worse overall survival in HNSC and LIHC. In conclusion, the above results provided a systematic analysis of aberrant calpain-mediate cleavage affected by missense variations and showed that calpain cleavage–related mutations were significantly involved in different cancers.

Although DeepCalpain has achieved promising performance, there is still room for improvement. First of all, the negative dataset is significantly larger than the positive dataset, which leads to a data unbalanced issue. Although we have tested a balanced dataset and it comes to a comparable performance compared with the existing model, more approaches need to be tried to overcome the issue (Umarov et al., 2019). It is well known that a larger training dataset will produce more accurate predictive performance. In the future, experimentally identified proteins with calpain cleavage sites will be continuously collected from the literature and integrated into the predictive model when available. Furthermore, with the development of high-throughput techniques, more calpain-specific cleavage sites will be identified, while the prediction systems could be generalized to other calpain isoforms besides μ-calpain and m-calpain. Thus, a powerful tool for the prediction of calpain cleavage sites in a calpain-specific fashion would be desirable. Furthermore, we will introduce some other features, such as secondary and three-dimensional structures, protein-protein interactions, and evolutionary information, into the prediction system in future developments. And more deep learning methods should be taken into consideration, such as CNN, recurrent neural network (RNN), graph convolutional neural network (GCN), and attention models (Li et al., 2019), which may help improve the current performance. Overall, we developed a powerful tool DeepCalpain for the identification of potential calpain cleavage sites with satisfying performance in this study. The systematic analysis of the connection between calpain cleavage and cancer mutations may help speed up our understanding of the regulatory mechanism of calpain-mediated cleavage in different cancer types and may open new avenues for the diagnosis and treatment of cancers.

## Data Availability

Publicly available datasets were analyzed in this study. This data can be found here: https://xenabrowser.net/datapages/.

## Author Contributions

HC and ZXL designed and supervised the experiments. ZXL, KY, JD, LZ, and ZKL performed experiments and data analysis, and developed the predictor. QZ, SL, and YD contributed to data analysis and predictor development. ZXL, KY, and HC wrote the manuscript with contributions of all the authors. All authors reviewed and revised the manuscript.

## Funding

This work was supported by grants from Program for Guangdong Introducing Innovative and Entrepreneurial Teams (2017ZT07S096 to ZXL), Pearl River S&T Nova Program of Guangzhou (201906010088 to ZXL), the National Natural Science Foundation of China (31601067 to HC, 81603019 to LZ), State Key Laboratory of Cotton Biology Open Funds to HC, the Jiangsu Provincial Natural Science Foundation, China (BK20150649 to LZ), and Sichuan Science and Technology Support Project (2018SZ0152 to JD).

## Conflict of Interest Statement

The authors declare that the research was conducted in the absence of any commercial or financial relationships that could be construed as a potential conflict of interest.

## Abbreviations

DeepCalpain, deep learning-based calpain cleavage sites prediction; GPS, Group-based prediction system; CNN, convolutional neural network; DNN, deep neural network; AAC, amino acid composition; CKSAAP, composition of k-spaced amino acid pairs; PSSM, position-specific scoring matrix; BE, binary encoding profiles; PSO, particle swarm optimization; Sn, sensitivity; Sp, specificity; Pr, precision; AUC, area under the ROC curve; ROC, receiver operating characteristic curve; Phylo-HMM, phylogenetic hidden Markov model; PPI, protein-protein interaction; CCRM, calpain cleavage related mutation; VAF, variant allele fraction; HNSC, head-neck squamous cell carcinoma; LIHC, liver hepatocellular carcinoma; RNN, recurrent neural network; GCN, graph convolutional neural network.

## Supplementary Material

Figure S1 The AUCs of different flanking residues around the cleavage sites.

Figure S2 The different mutation pattern between CCRMs and nCCRMs. **(A)** Counting for the different types of mutation for CCRMs and nCCRMs. **(B)** The cumulative distribution function for the VAFs of CCRMs and nCCRMs.

Table S1 From the literature and public data resources, we totally curated 442 experimentally identified calpain cleavage sites in 169 proteins, which contained 176 µ-calpain cleavage sites and 256 m-calpain cleavage sites.

Table S2 The AUC values of random flanking sites around the central motif.

Table S3 The hyperparameters and model structure of DeepCalpain adjusted by PSO algorithm.
