# Supplementary figures and images for: Precise Prediction of Calpain Cleavage Sites and Their Aberrance Caused by Mutations in Cancer
Source: Front Genet. 2019 Aug 8;10:715. doi: 10.3389/fgene.2019.00715 (PMC6694742; doi:10.3389/fgene.2019.00715)

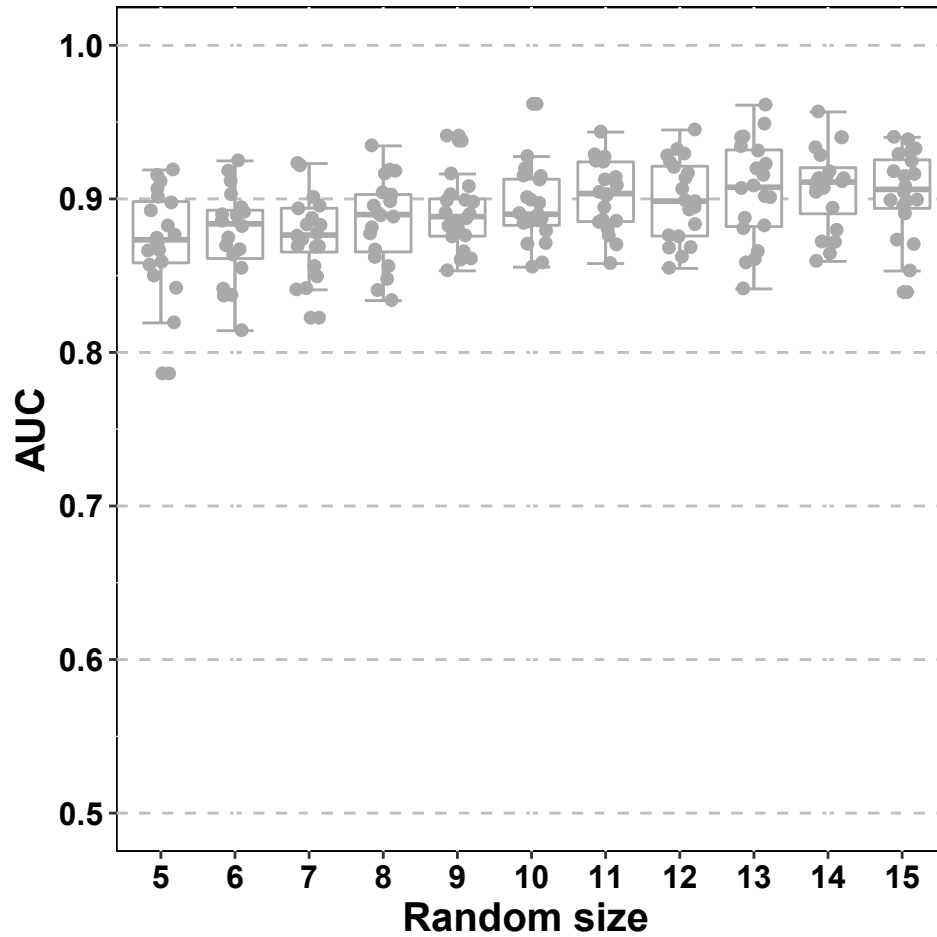

Figure S1. The AUCs of different flanking residues around the cleavage sites.

Supplement: Figure S1 — The AUCs of different flanking residues around the cleavage sites. [file Image_1.pdf]
