# Supplementary material for: Precise Prediction of Calpain Cleavage Sites and Their Aberrance Caused by Mutations in Cancer
Source: Front Genet. 2019 Aug 8;10:715. doi: 10.3389/fgene.2019.00715 (PMC6694742; doi:10.3389/fgene.2019.00715)
Supplement: Figure S2 — The different mutation pattern between CCRMs and nCCRMs. (A) Counting for the different types of mutation for CCRMs and nCCRMs. (B) The cumulative distribution function for the VAFs of CCRMs and nCCRMs. [file Image_2.pdf]

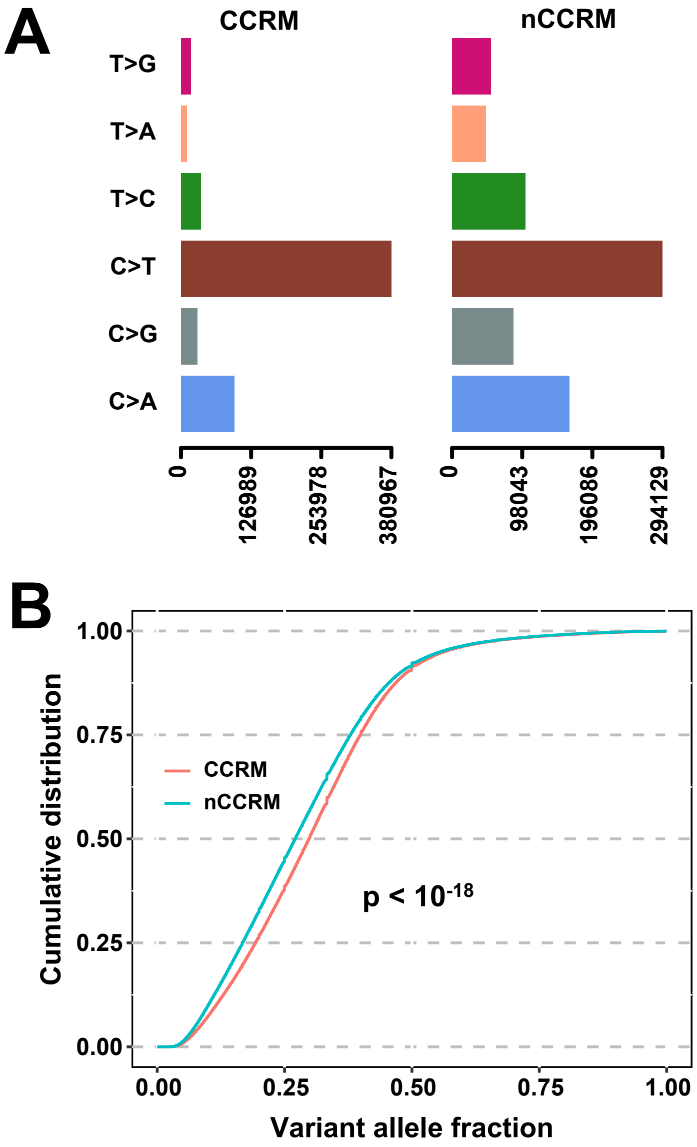

Figure S2. The different mutation pattern between CCRMs and nCCRMs. (A) Counting for the different types of mutation for CCRMs and nCCRMs. (B) The cumulative distribution function for the VAFs of CCRMs and nCCRMs.
